# The relationship between peptic ulcer and cardiovascular disease in elderly population: a study on mortality and disease development

**DOI:** 10.3389/fmed.2025.1610867

**Published:** 2025-08-26

**Authors:** Lin He, Qianlei Wang, Yang Qiu, Nan Shen, Peimin Pu, Ruiqing Wang, Hongyu Miao, Haiyan Zhang, Xiao Yu, Dinghong Xiao, Lianjun Xing, Zhidong Liu

**Affiliations:** ^1^Longhua Hospital Shanghai University of Traditional Chinese Medicine, Shanghai, China; ^2^Shanghai University of Traditional Chinese Medicine, Shanghai, China

**Keywords:** peptic ulcer, cardiovascular disease, elderly population, comorbidity research, mortality risk

## Abstract

**Background:**

Peptic ulcer (PU) and cardiovascular disease (CVD) are significant chronic illnesses, particularly in the elderly. This study investigates the relationship between PU and CVD in older adults and the impact on mortality risk.

**Methods:**

This study was conducted utilizing data from a nationwide health survey of the elderly in China. Kaplan–Meier curves and log-rank tests were applied in survival analysis to evaluate mortality differences between the groups. Stratified models were applied to evaluate the effects of factors.

**Results:**

This study included 3,636 participants. CVD was significantly associated with an increased PU risk (OR = 1.31, 95%CI 1.03–1.66, *p* = 0.04), while PU had no significant effect on CVD incidence (OR = 1.08, 95%CI 0.77–1.51, *p* = 0.64). Mortality risk was significantly higher in the CVD group (HR = 1.22, 95%CI 1.03–1.45, *p* = 0.02) compared to the non-comorbid group. No significant difference in mortality was observed between the PU-only and combined PU-CVD groups. Stratified analysis identified advanced age (≥75 years) (HR = 1.45, 95%CI 1.06–1.87, *p* < 0.01) and male gender (HR = 1.29, 95%CI 1.05–1.62, *p* < 0.01) as significant mortality risk factors among PU patients.

**Conclusion:**

PU does not have a significant impact on overall mortality or the prognosis of CVD patients. CVD was a risk factor for PU, but PU did not significantly increase CVD risk. A higher mortality risk was observed in older and male PU patients. These findings suggest the need for gender-sensitive and age-stratified management strategies for PU in high-risk groups.

## Graphical abstract

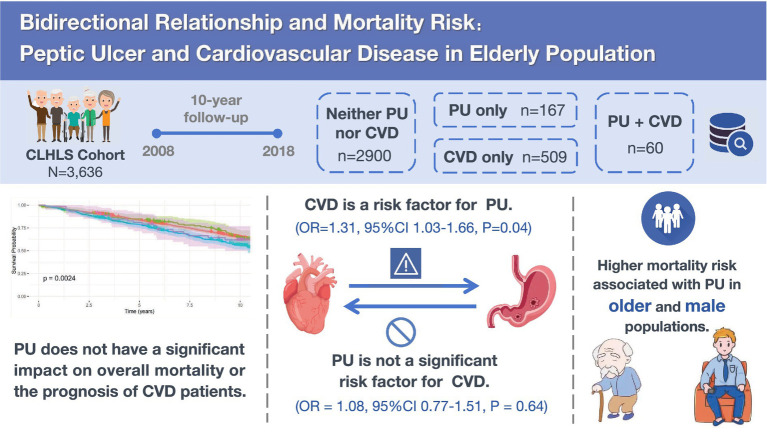

## 1 Introduction

Peptic ulcer (PU) and cardiovascular disease (CVD) are significant chronic illnesses, especially among the elderly, due to their comorbidity and combined impact on mortality (1). PU involves the formation of ulcers in the mucosal lining of the digestive tract, particularly in the lower esophagus, stomach, or duodenum, which may extend through the mucosal layer into the underlying submucosal tissues (2). PU affects around 4 million people annually, with a global lifetime prevalence of 5–10%. The incidence varies from 0.3 to 0.9 per thousand individuals annually (3), while around 54,000 hospitalizations annually in the U.S. are linked to PU bleeding (4). In mainland China, precise national PU prevalence is unknown, though a 2007 Shanghai survey with 1,022 participants found gastric ulcers in 6.07% and duodenal ulcers in 13.31% (5). Key risk factors for PU include *Helicobacter pylori* (*H. pylori*) infection, nonsteroidal anti-inflammatory drugs (NSAIDs) use, aging, smoking and alcohol (3). Age increases PU risk and complications (6, 7). Despite treatment options like acid suppressants, *H. pylori* eradication, lifestyle changes, and NSAIDs management, PU leads to around 10,000 deaths in the United States annually (4). Consequently, PU continues to be a critical health concern, especially for the elderly.

CVD stands as one of the most challenging global public health burdens, encompassing a range of complex pathological conditions resulting from cardiac and circulatory dysfunction. The clinical manifestations of CVD encompass arrhythmias, heart failure, coronary heart disease and stroke (8, 9). Annually, CVD accounts for approximately 17.9 million deaths, and its global incidence has doubled over the past two decades (10), representing one-third of total global mortality (11). In China, CVD remains the primary contributor to both mortality and premature death (12), contributing to 40% of the national mortality rate (13). The burden of CVD is particularly significant among the elderly population (14), often accompanied by comorbidities and risk factors such as obesity, hypertension, diabetes, dyslipidemia, and smoking (15).

Previous studies have demonstrated a multidimensional interaction between PU and CVD, including drug-induced damage, overlapping risk factors, and inflammatory pathway synergy. In patients with CVD, antiplatelet drugs such as aspirin elevate the risk of developing PU. Smoking and advanced age are common risk factors for both PU and CVD. *Helicobacter pylori* infection, a common cause of PU, is positively correlated with CVD through virulence factors (16), and its eradication reduces CVD risk (17). The pathophysiological mechanisms of both diseases may involve shared pathways of oxidative stress and immune modulation (18, 19). While the relationship between PU and CVD is complex, the precise link between the two and the influence of comorbidities on mortality remains unclear. Some studies suggest that PU increases CVD risk through systemic inflammation and endothelial dysfunction, with a UK Biobank study of 330,751 individuals showing higher CVD incidence among PU patients (19). However, other research indicates that PU may have a protective role or negligible impact on CVD prognosis. A meta-analysis of 230,288 patients found that HP infection slightly increases cardiovascular risk, far less than earlier studies suggested (16). These inconsistencies highlight the need for further research to clarify the interaction between these diseases. This study uses data from the Chinese Longitudinal Healthy Longevity Survey (CLHLS) database spanning from 2008 to 2018 to explore the relationship between PU and CVD, with a focus on mortality impact and the potential modifying role of demographic and clinical factors.

## 2 Methods

### 2.1 Study design

The study utilized data from the CLHLS, a nationwide cohort of older adults in China, aimed at identifying determinants of healthy longevity among those aged 79 and above, and 65–79. The CLHLS is conducted in 23 of China’s 31 provinces, covering 85.0% of the population. Data are collected through home interviews with participants, carried out by trained interviewers in randomly selected counties and cities (20). The study adheres to the Declaration of Helsinki and has received approval from the Peking University Ethics Committee (IRB00001052-13074). All participants provided informed consent. We analyzed follow-up data from four time points: 2008, 2011, 2014, and 2018. The baseline survey in 2008 included 16,954 respondents, who were then followed up at the three subsequent time points.

**Figure 1:**
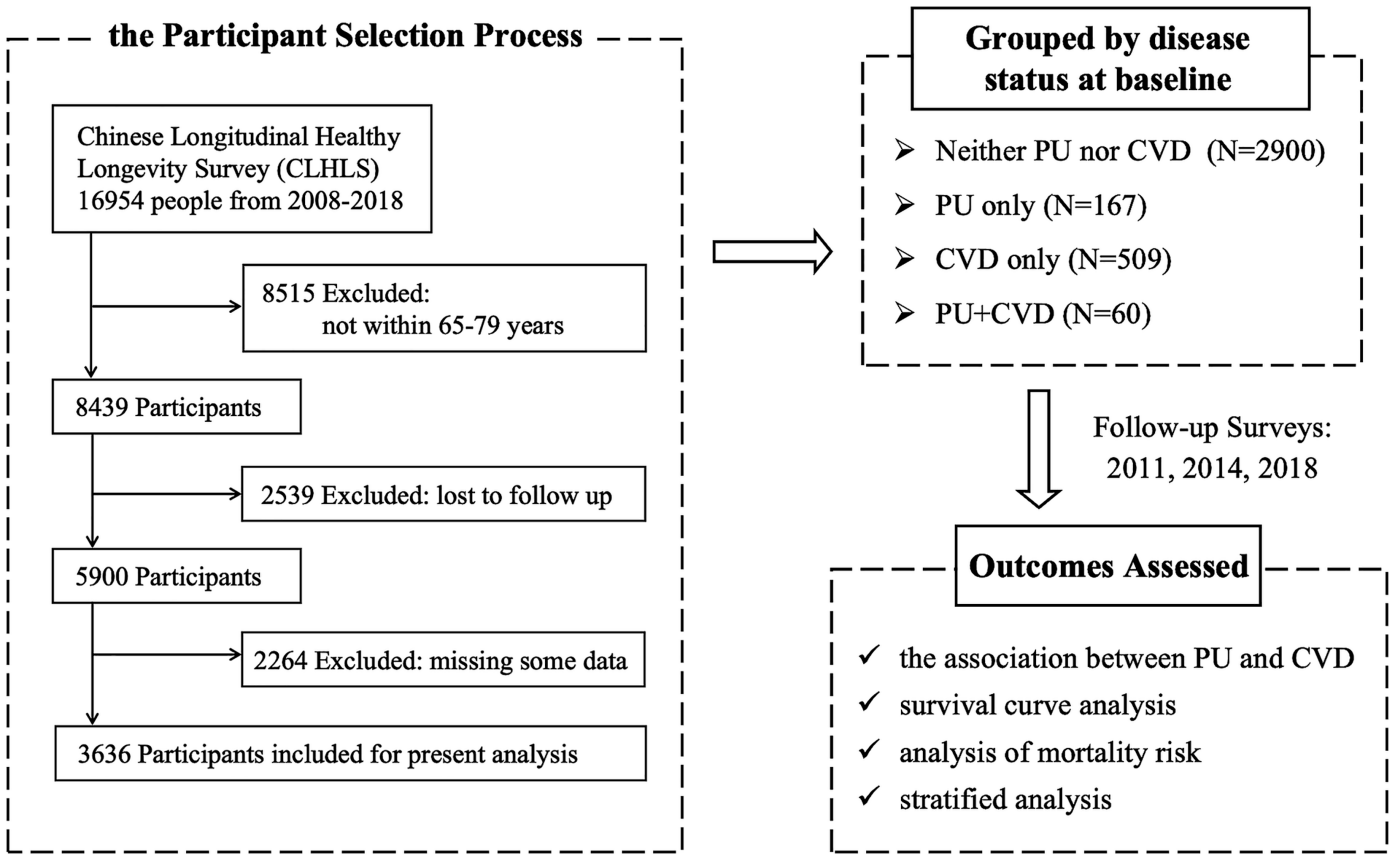
Methodological flowchart summarizing the study design and procedures of the CLHLS cohort from 2008 to 2018.

Participants were required to meet the following inclusion criteria: (1) baseline age between 65 and 79 years, and (2) complete diagnostic information on PU and CVD at both baseline and follow-up assessments. The exclusion criteria were as follows: (1) loss to follow-up, and (2) missing critical information at baseline or during follow-up assessments, including demographic data, health behaviors, or comorbid conditions (Figure 1).

### 2.2 Diagnostic of PU and CVD

The primary outcomes of this study are survival data and the incidence of PU and CVD. PU includes gastric and duodenal ulcers, while CVD comprises heart disease, stroke, and cerebrovascular diseases. The identification of PU and CVD is based on responses to question G14 of the CLHLS questionnaire, ‘Do you currently have any of the following chronic diseases?’ To minimize reporting bias, we used a stratified diagnostic approach: (1) individuals indicating both ‘disease present’ and ‘hospital-diagnosed’ are classified as suspected cases; (2) persistent reporting of the condition in subsequent follow-ups is required for confirmation, otherwise, they are excluded from the confirmed group; (3) cases reported only once during follow-up without supporting data are provisionally classified as diseased.

Additional covariates include demographic and health factors. Demographic factors encompass sex, age, body mass index (BMI), ethnicity (Han or others), education years, marital status, income, residential area (urban or rural), and living arrangement (with or without family). Health variables include self-reported health status, quality of life, smoking, alcohol use, physical activity, and medical conditions such as hypertension, diabetes, cancer, and arthritis.

### 2.3 Survival data and censoring

This study obtained mortality information through multiple sources to ensure data reliability. First, statutory death registration data provided by local health authorities were integrated, which included household deregistration records and death certificates issued by medical institutions. Second, a dynamic monitoring system was established, where trained research teams conducted regular home visits to track participants’ survival status in real time. In cases without official death records, a standardized tracing procedure was implemented by verifying information through interviews with the deceased’s immediate family members or informed neighbors. For participants lost to follow-up, structured phone interviews were conducted to confirm survival outcomes. All data were subjected to a three-tier quality control system: (1) cross-referencing original data from multiple sources; (2) automatic screening of logical discrepancies; (3) final review by an independent committee of epidemiologists and clinicians to ensure the validity and reliability of mortality determination.

In this study, participants’ survival data were recorded from the baseline in 2008 until the last follow-up in 2018. For participants lost to follow-up, data were considered right-censored, with the censoring time extending from the baseline interview date to the last available follow-up date. Survival time was defined as the period from baseline to death or censoring.

### 2.4 Statistical analysis

Descriptive statistics were applied to present the baseline characteristics of the participants. Continuous variables were expressed as mean ± standard deviation (SD), while categorical variables were represented as counts and percentages. Comparisons between groups were performed using ANOVA and chi-square tests. To assess the association between PU and CVD incidence, multivariable logistic regression models were applied, with odds ratios (OR) and 95% confidence intervals (CI) calculated after adjusting for potential confounders.

Stratified analysis and interaction terms were employed to explore subgroup heterogeneity. Kaplan–Meier curves and log-rank test compared survival times, while Cox models calculated hazard ratios (HR) and 95%CI for mortality risk. Multivariable adjustments were made in both logistic and Cox models. Model 1 was the crude model. Model 2 adjusts for key demographic variables. Model 3 further includes health behavior indicators and comorbidity status to control for additional confounding effects. Stratified analyses were performed to evaluate the mortality risk associated with PU across various subpopulations, using sex, age, BMI, arthritis, and CVD status as stratification factors. Subgroup stratification was conducted to further explore potential effect modifiers. BMI was included to distinguish whether the observed association between CVD and PU was independent of nutritional status. Age and sex were selected given their fundamental relevance to both disease prevalence and clinical outcomes. Arthritis was included as a proxy indicator for NSAID exposure, as patients with arthritis commonly use NSAIDs, and direct medication data were not available in the CLHLS database.

All statistical analyses were conducted using SPSS version 26.0 (IBM, Chicago, IL, USA) and R version 4.2.1 (R Foundation for Statistical Computing). A *p*-value below 0.05 was considered indicative of statistical significance.

## 3 Results

### 3.1 Characteristics of study population

A total of 3,636 participants, aged 65–80 years, were included in this study (Table 1), with a median follow-up duration of 8.52 years. The average age of the participants was 70.96 years, with 1,935 males (53.21%) and 1,701 females (46.79%). Among the participants, 93.70% were of Han ethnicity, and more than half had a moderate economic status (71.34%). Over half of the participants had a BMI within the normal range (58.25%). Most participants lived in rural areas (88.14%) and cohabited with family members (85.72%). In terms of lifestyle behaviors, 43.98% of participants had a history of smoking, 37.57% had a history of alcohol consumption, and 39.77% had a habit of physical exercise.

**Table 1:** Baseline characteristics of participants included in the CLHLS cohort from 2008 to 2018.

Characteristic	Neither PU nor CVD	PU only	CVD only	PU + CVD	*p-*value
	(*N* = 2,900)	(*N* = 167)	(*N* = 509)	(*N* = 60)	
Age					
Mean (SD)	72.1 (4.20)	71.5 (3.85)	72.4 (4.31)	71.9 (4.23)	0.112
Median [Min, Max]	72.0 [65.0, 79.0]	71.0 [65.0, 79.0]	73.0 [65.0, 79.0]	72.0 [65.0, 79.0]	
Sex (male)	1,566 (54.0%)	92 (55.1%)	245 (48.1%)	32 (53.3%)	0.101
Ethnic (Han Chinese)	2,702 (93.2%)	155 (92.8%)	492 (96.7%)	58 (96.7%)	0.094
Education					
Educated	1,687 (58.2%)	96 (57.5%)	325 (63.9%)	42 (70.0%)	0.030
Illiteracy	1,213 (41.8%)	71 (42.5%)	184 (36.1%)	18 (30.0%)	
Economic status					
Excellent	28 (1.0%)	1 (0.6%)	7 (1.4%)	1 (1.7%)	0.485
Good	342 (11.8%)	14 (8.4%)	60 (11.8%)	6 (10.0%)	
Average	2076 (71.6%)	123 (73.7%)	359 (70.5%)	36 (60.0%)	
Poor	393 (13.6%)	26 (15.6%)	69 (13.6%)	15 (25.0%)	
Very poor	61 (2.1%)	3 (1.8%)	14 (2.8%)	2 (3.3%)	
Marriage					
Married (cho.)	1890 (65.2%)	110 (65.9%)	342 (67.2%)	46 (76.7%)	0.199
Married (sep.)	98 (3.4%)	7 (4.2%)	8 (1.6%)	1 (1.7%)	
Divorced	10 (0.3%)	0 (0%)	4 (0.8%)	1 (1.7%)	
Widowed	866 (29.9%)	49 (29.3%)	151 (29.7%)	12 (20.0%)	
Never married	36 (1.2%)	1 (0.6%)	4 (0.8%)	0 (0%)	
Birth place					
Urban	296 (10.2%)	22 (13.2%)	103 (20.2%)	10 (16.7%)	<0.001
Rural	2,604 (89.8%)	145 (86.8%)	406 (79.8%)	50 (83.3%)	
Co-residence					
Living with family	2,471 (85.2%)	141 (84.4%)	451 (88.6%)	54 (90.0%)	0.345
Living alone	412 (14.2%)	26 (15.6%)	55 (10.8%)	6 (10.0%)	
Residential care facility	17 (0.6%)	0 (0%)	3 (0.6%)	0 (0%)	
Self-reported QoL					
Excellent	418 (14.4%)	21 (12.6%)	74 (14.5%)	9 (15.0%)	<0.001
Good	1,270 (43.8%)	60 (35.9%)	193 (37.9%)	17 (28.3%)	
Fair	1,037 (35.8%)	75 (44.9%)	203 (39.9%)	23 (38.3%)	
Poor	158 (5.4%)	10 (6.0%)	33 (6.5%)	11 (18.3%)	
Very poor	17 (0.6%)	1 (0.6%)	6 (1.2%)	0 (0%)	
Self-reported health					
Excellent	434 (15.0%)	18 (10.8%)	48 (9.4%)	3 (5.0%)	<0.001
Good	1,174 (40.5%)	47 (28.1%)	121 (23.8%)	12 (20.0%)	
Fair	922 (31.8%)	68 (40.7%)	166 (32.6%)	19 (31.7%)	
Poor	341 (11.8%)	32 (19.2%)	155 (30.5%)	24 (40.0%)	
Very poor	29 (1.0%)	2 (1.2%)	19 (3.7%)	2 (3.3%)	
Smoking					
Current	810 (27.9%)	49 (29.3%)	95 (18.7%)	17 (28.3%)	<0.001
Never	1,638 (56.5%)	88 (52.7%)	283 (55.6%)	28 (46.7%)	
Former	452 (15.6%)	30 (18.0%)	131 (25.7%)	15 (25.0%)	
Drinking					
Current	713 (24.6%)	43 (25.7%)	64 (12.6%)	12 (20.0%)	<0.001
Never	1811 (62.4%)	99 (59.3%)	326 (64.0%)	34 (56.7%)	
Former	376 (13.0%)	25 (15.0%)	119 (23.4%)	14 (23.3%)	
Exercising	1,105 (38.1%)	57 (34.1%)	253 (49.7%)	31 (51.7%)	<0.001
BMI group					
<18.5	548 (18.9%)	37 (22.2%)	70 (13.8%)	9 (15.0%)	<0.001
18.5–23.9	1732 (59.7%)	109 (65.3%)	241 (47.3%)	36 (60.0%)	
24–27.9	490 (16.9%)	19 (11.4%)	153 (30.1%)	13 (21.7%)	
>28	130 (4.5%)	2 (1.2%)	45 (8.8%)	2 (3.3%)	
Base hypertension	561 (19.3%)	26 (15.6%)	252 (49.5%)	32 (53.3%)	<0.001
Base diabetes	89 (3.1%)	4 (2.4%)	54 (10.6%)	3 (5.0%)	<0.001
Base cancer	13 (0.4%)	1 (0.6%)	4 (0.8%)	0 (0%)	0.720
Base arthritis	659 (22.7%)	57 (34.1%)	129 (25.3%)	31 (51.7%)	<0.001
Follow-up duration					
Mean (SD)	7.33 (3.06)	7.35 (3.01)	6.69 (3.11)	7.19 (3.20)	<0.001
Median [Min, Max]	8.59 [0.00821, 11.1]	8.79 [0.0192, 11.1]	6.07 [0.101, 11.1]	7.92 [0.320, 11.1]	

CVD, cardiovascular disease; PU, peptic ulcer; QoL, quality of life; SD, standard deviation; Min, minimum; Max, maximum.

At baseline, 227 participants were diagnosed with PU, and 569 were diagnosed with CVD. Based on the presence of PU or CVD, the total population was divided into four groups: 2,900 participants (79.76%) with neither PU nor CVD, 167 participants (4.59%) with only PU, 509 participants (14.0%) with only CVD, and 60 participants (1.65%) with both PU and CVD. We found that more CVD patients were from urban areas, while more PU patients were from rural areas, suggesting a potential association between the occurrence of these diseases and regional economic conditions. Regarding lifestyle habits, more CVD patients had a history of smoking, while more PU patients had a history of alcohol consumption, which aligns with the primary risk factors for CVD and PU. The majority of PU and CVD patients did not engage in physical activity, indicating room for improvement in their lifestyles. In terms of health status, PU patients had a lower BMI and poorer self-rated health and quality of life, but they had a longer survival time.

### 3.2 Analysis of the association between PU and CVD

**Figure 2:**
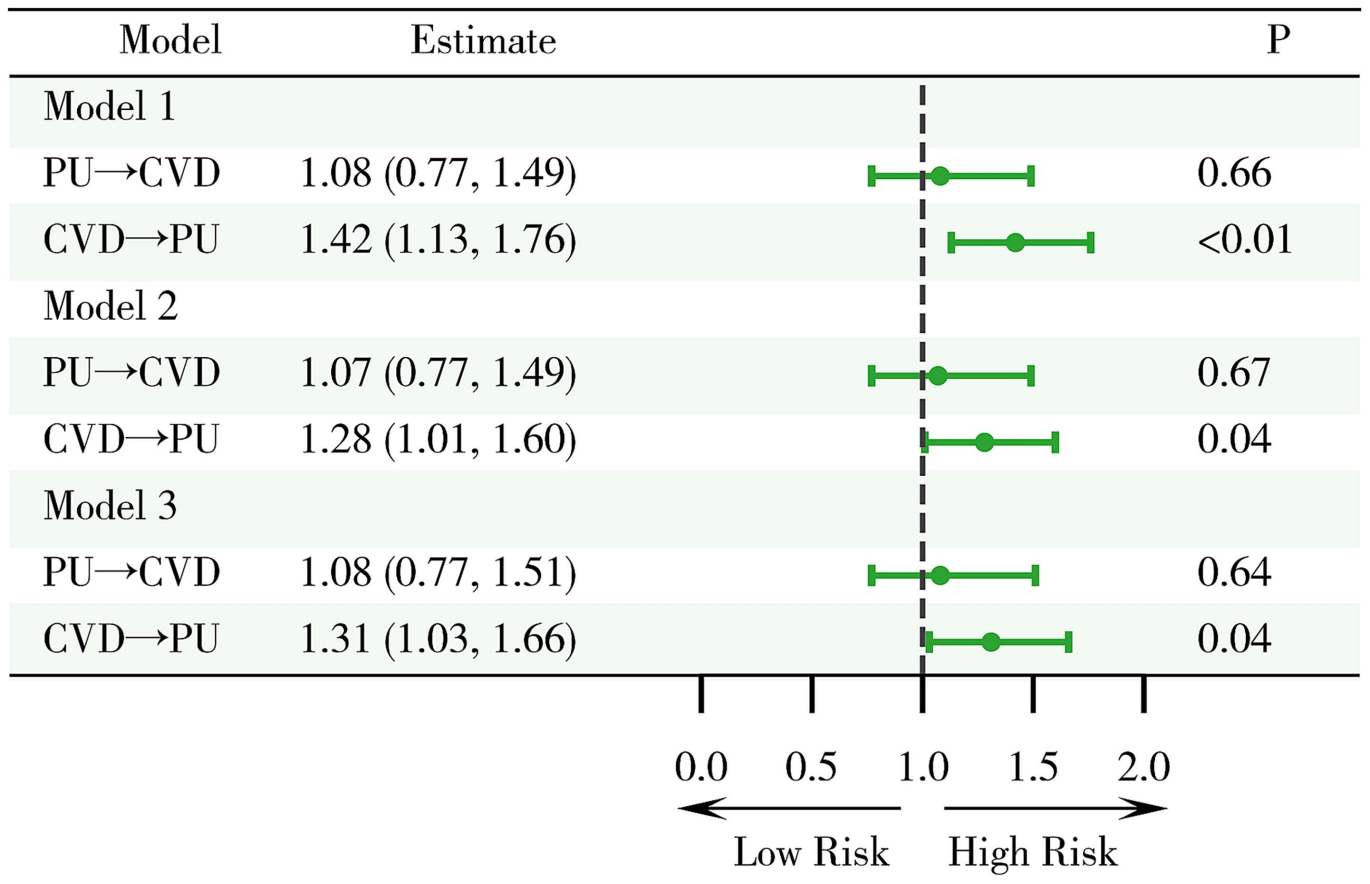
Examination of the relationship between PU and cardiovascular disease CVD. Model 1: crude model; Model 2: adjusted for key demographic variables. Model 3: adjusted for key demographic variables, health behavior indicators, and comorbidity status. CVD, cardiovascular disease; PU, peptic ulcer.

This study systematically analyzed the association between PU and CVD (Figure 2). The results indicated that at baseline, among patients without CVD, those with PU did not show a statistically significant increased risk of developing CVD in the future compared to those without PU (OR = 1.08, 95%CI 0.77–1.49, *p* = 0.66). This lack of significant difference persisted even after adjustments in multivariable models (Model 2: OR = 1.07, 95% CI 0.77–1.49, *p* = 0.67; Model 3: OR = 1.08, 95%CI 0.77–1.51, *p* = 0.64), indicating that PU does not significantly increase the risk of CVD.

On the other hand, among patients without PU at baseline, CVD was found to be a significant risk factor (OR = 1.42, 95%CI 1.13–1.76, *p* < 0.01), a finding consistent across multiple models (Model 2: OR = 1.28, 95%CI 1.01–1.60, *p* = 0.04; Model 3: OR = 1.31, 95%CI 1.03–1.66, *p* = 0.04). This suggests that CVD is a relatively robust risk factor for the development of PU.

In summary, the study findings suggest that CVD has a significant impact on the occurrence of PU, whereas PU does not significantly affect the risk of developing CVD.

### 3.3 Analysis of mortality risk

**Figure 3:**
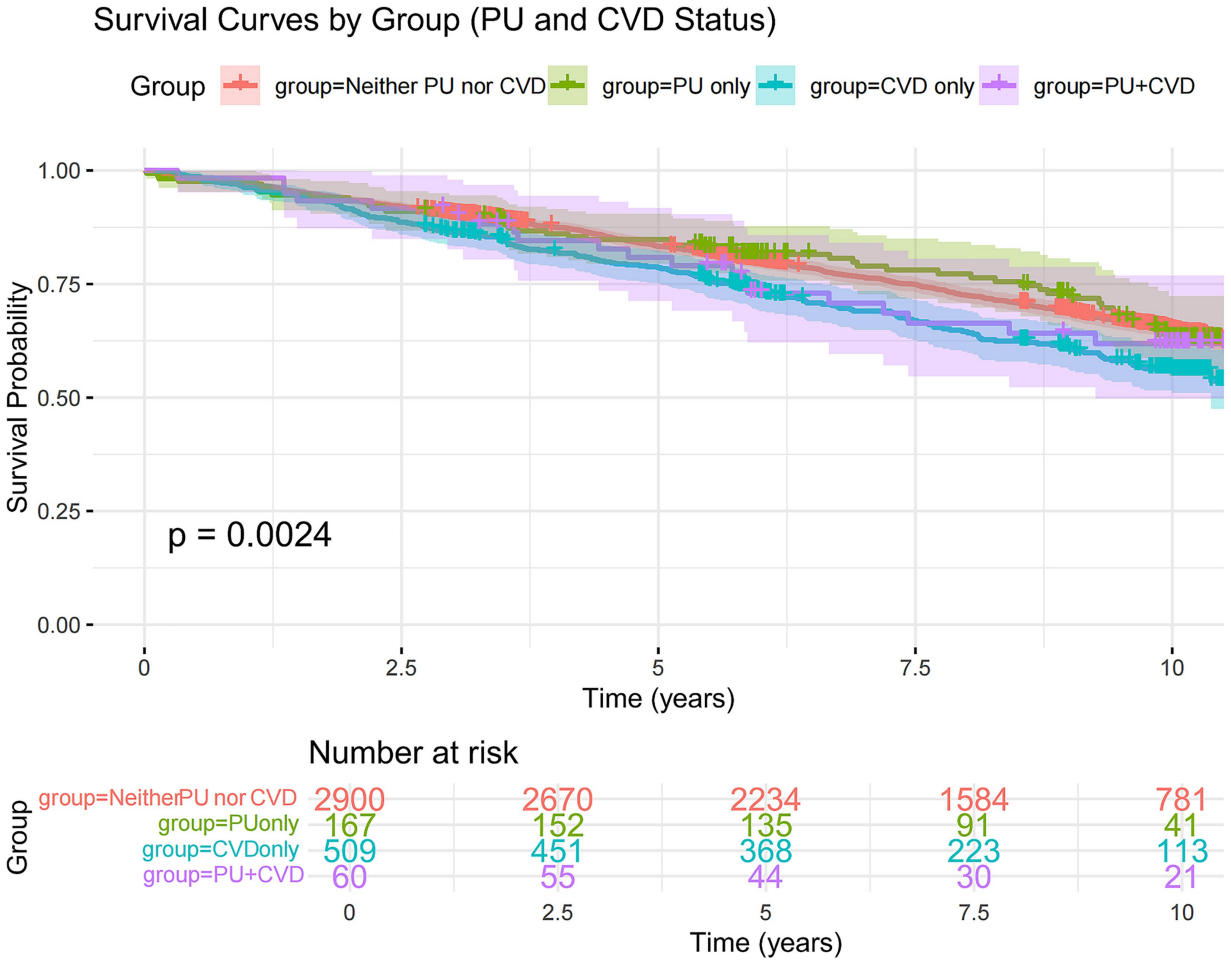
Kaplan–Meier curve of survival probability over time, stratified by PU and CVD status. The solid lines represent the survival curves, and the shaded areas indicate the 95% CIs. Vertical lines denote the transplant-free survival probabilities at 5 and 10 years for each group: participants with neither PU nor CVD (red), PU only (green), CVD only (blue), and both PU and CVD (purple). CVD, cardiovascular disease; PU, peptic ulcer.

Survival curve analysis of the four patient groups indicated that, compared to those without PU or CVD, patients with CVD experienced higher mortality rates (Figure 3) while no significant increase in mortality was observed in those with PU alone. This suggests that CVD is indeed an important risk factor for mortality, whereas PU does not have a significant impact on death. Notably, PU may serve as a protective factor or have no effect on CVD.

**Figure 4:**
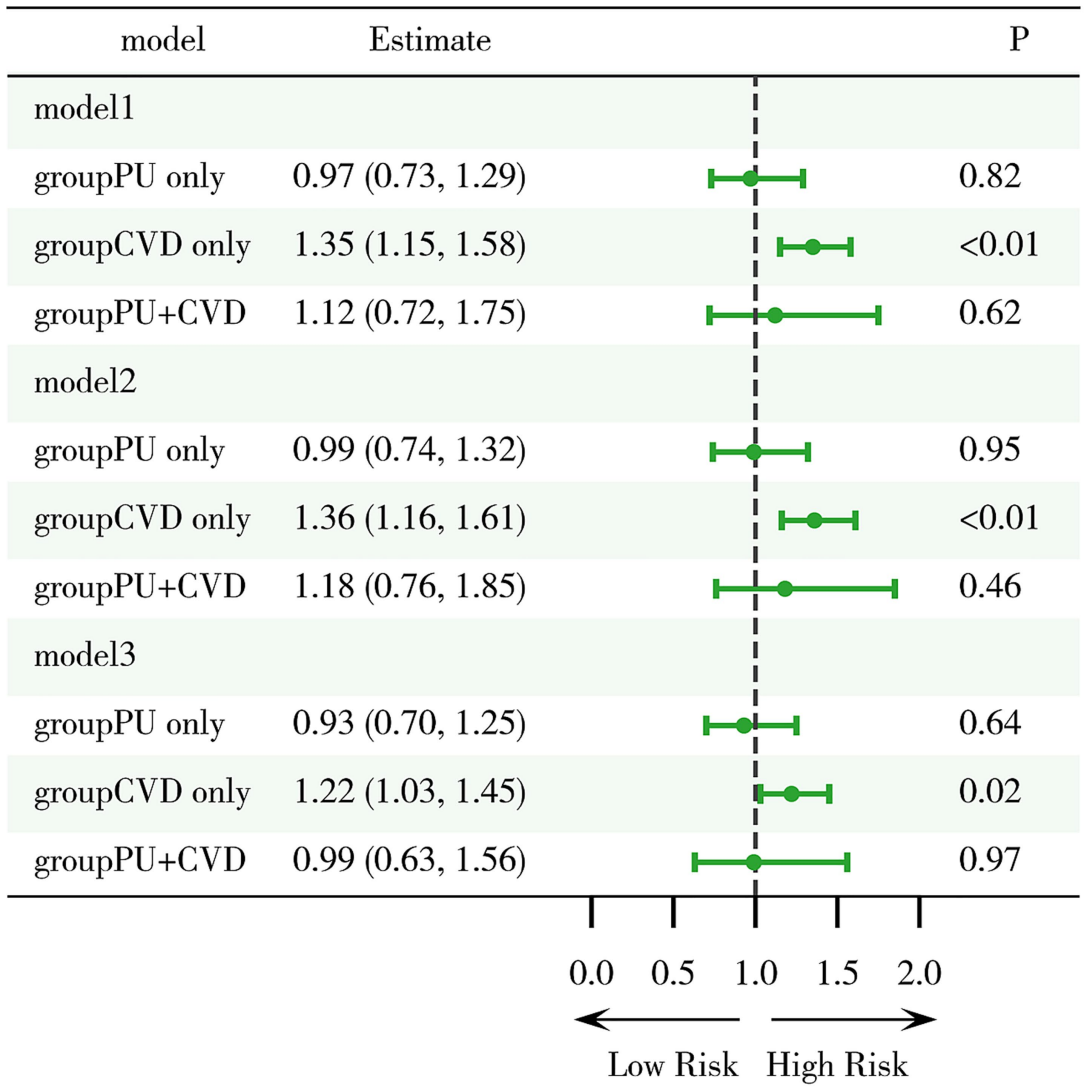
Evaluation of mortality risk associated with PU and CVD. Model 1: crude model; Model 2: adjusted for key demographic variables. Model 3: adjusted for key demographic variables, health behavior indicators, and comorbidity status. CVD, cardiovascular disease; PU, peptic ulcer.

Further analysis of mortality risk across different models revealed that, compared to the group with neither PU nor CVD, the mortality risk in the only CVD group remained significantly higher (Model 1: HR = 1.35, 95%CI 1.15–1.58, *p* < 0.01; Model 2: HR = 1.36, 95%CI 1.16–1.61, *p* < 0.01; Model 3: HR = 1.22, 95%CI 1.03–1.45, *p* = 0.02) (Figure 4). However, the mortality risk in the only PU group and the PU with CVD group was not significantly different across all three models. Moreover, the interaction between CVD and PU was negative (*p* = 0.28). These findings suggest that PU itself does not have a significant effect on mortality, nor does it substantially influence the mortality risk in CVD patients.

**Figure 5:**
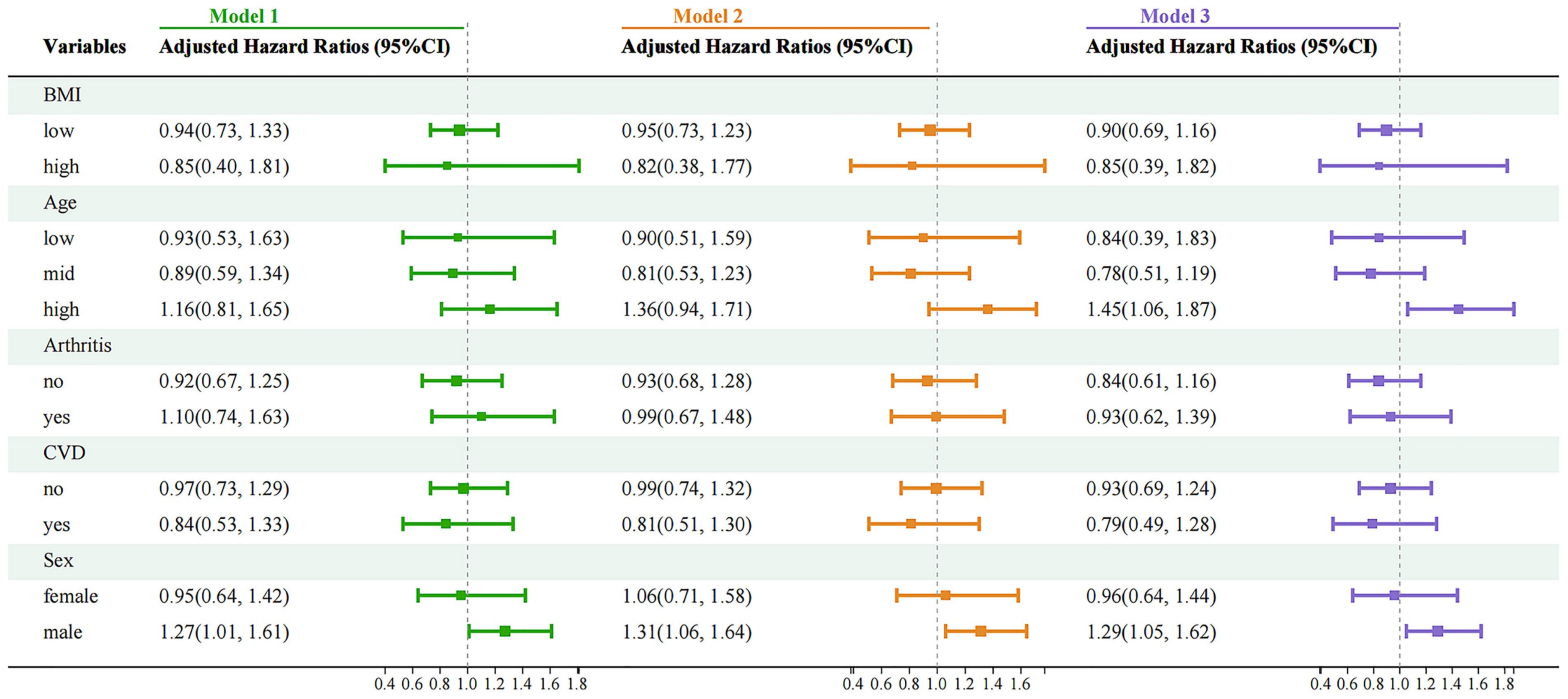
Stratified analysis of mortality risk associated with PU and CVD across different subgroups. Model 1: crude model; Model 2: adjusted for key demographic variables. Model 3: adjusted for key demographic variables, health behavior indicators, and comorbidity status. CVD, cardiovascular disease; PU, peptic ulcer.

To further explore the impact of PU on mortality risk across different subgroups, a stratified analysis was conducted based on five factors: BMI, age, sex, CVD status and history of arthritis (Figure 5). The results indicated a significantly increased mortality risk among older adults (Model 3: HR = 1.45, 95%CI 1.06–1.87, *p* < 0.01) and males (Model 3: HR = 1.29, 95%CI 1.05–1.62, *p* < 0.01), whereas no significant associations were observed in other strata. These findings suggest that older adults and males may be at higher risk and should be particularly cautious regarding the potential health implications of PU. It is recommended that primary and secondary prevention measures be selectively implemented in these high-risk groups to improve outcomes and benefit patients.

## 4 Discussion

This study thoroughly explored the relationship between PU and CVD in the elderly population in China. The results indicated that PU does not have a significant impact on overall mortality or the prognosis of CVD patients. Additionally, CVD was found to be a risk factor for the occurrence of PU, while PU was not identified as a significant risk factor for the development of CVD. However, stratified analysis revealed an association between PU and higher mortality risk in older and male populations. These findings suggest that although PU may not be a significant prognostic factor in the general population, it warrants greater attention in high-risk subgroups, highlighting the need for gender-sensitive approaches and age-stratified management strategies for PU.

The study found that PU did not have a significant impact on overall mortality or the prognosis of CVD patients. Research indicates that the burden of PU in China has shown a downward trend (21), primarily due to the nationwide implementation of standardized *H. pylori* screening and eradication programs. PU patients, having previously experienced ulcers, often become more aware of the risks related to diet and medication, which may explain why PU does not significantly impact overall mortality. Although our study found no significant association between PU and overall mortality in patients with CVD, the potentially fatal nature of PUD should not be overlooked. Acute complications such as bleeding and perforation often carry high short-term mortality. These events may be especially dangerous in CVD patients due to reduced cardiovascular reserve (23), underscoring the need for early recognition and proactive management in high-risk populations.

Furthermore, our study highlights that the impact of PU on mortality varies by sex and age, with males and older adults at a higher risk. This is consistent with a global study on the burden of PU conducted from 1990 to 2009 across 204 countries and regions (23), which revealed that the highest prevalence, mortality, and disability-adjusted life years (DALYs) for PU occurred in the 80–84 and 85+ age groups. In these age groups, the mortality rate increased sharply, likely due to the decline in gastrointestinal mucosal barrier function and impaired repair capacity in elderly individuals, as well as the increased use of NSAIDs due to comorbid chronic conditions (24). Furthermore, our study found that the mortality risk associated with PU is higher in men. This may be due to the protective effect of estrogen on the gastrointestinal mucosa, which promotes increased bicarbonate secretion and enhances phospholipid levels (25). Additionally, men tend to be more exposed to complex risk factors, such as overeating and excessive alcohol intake, which not only further impact their health and mortality but also reflect difficult-to-overcome unhealthy lifestyle habits, particularly under high societal pressure. These factors may contribute to the higher mortality observed in male PU patients.

As a risk factor for PU, CVD may be primarily associated with the widespread use of NSAIDs and antiplatelet drugs in its treatment. NSAIDs decrease prostaglandin synthesis through the inhibition of COX-1/COX-2, resulting in reduced mucosal blood flow, disruption of the mucosal barrier, and impaired epithelial regeneration, while also directly inducing mitochondrial oxidative phosphorylation uncoupling and intestinal cell damage (26, 27). Non-NSAIDs antiplatelet drugs, such as clopidogrel, inhibit platelet-derived growth factor, which hinders ulcer healing (28). Ticagrelor, a commonly used antiplatelet agent, increases bleeding risk via epigenetic mechanisms. GPD2 hypermethylation suppresses ROS production and NF-κB-mediated P2Y12 expression, impairing platelet reactivity and enhancing susceptibility to ticagrelor-associated gastrointestinal bleeding (32). Additionally, impaired neural regulation between the central nervous system and the gastrointestinal tract in patients with cerebral infarction, including stress responses, parasympathetic excitation, and noradrenergic activation, may contribute to mucosal damage and increase the risk of gastrointestinal bleeding (22).

In this study, PU was not found to be a major determinant in the onset of CVD. PU is often associated with CVD through factors such as *H. pylori* infection, proton pump inhibitors (PPIs) use, smoking, and other harmful lifestyle habits, Certain studies have indicated that *H. pylori* infection may facilitate the development of atherosclerotic plaques, thereby increasing the risk of CVD (31), while PPIs may increase CVD risk through mechanisms like cellular damage, adverse drug interactions, and disruptions in metabolic processes (26). However, these studies tend to have small sample sizes and limited impact, so their findings may not be broadly applicable. A large-scale study with 23,000 participants and a median follow-up of 6.3 years found that the effect of *H. pylori* on CVD risk was minimal (27). Additionally, a meta-analysis showed that while PPIs use reduced the risk of gastrointestinal events, it did not significantly increase major cardiovascular adverse events, including cardiovascular-related deaths, all-cause mortality, or nonfatal myocardial infarctions (32). These studies support our finding that PU is not a significant risk factor for CVD, aligning with the results of our research. Furthermore, most existing studies have focused on the role of mediators such as *H. pylori* and PPIs in the relationship between PU and CVD, with fewer directly examining the connection between PU and CVD itself. This opens up new avenues for future research.

This study boasts several key strengths, such as the inclusion of a large, well-defined cohort derived from the CLHLS, complemented by longitudinal follow-up data extending over a decade. We employed robust multivariable adjustment models and stratified analysis methods, which enhanced the reliability of our findings. The non-significant interaction between PU and CVD (*p* = 0.28) suggests that their respective effects on mortality are likely independent rather than synergistic. However, this result may be partly attributed to the limited number of participants with both conditions, which reduced the statistical power to detect interaction effects. These insights reinforce the importance of early detection and preventive strategies in high-risk populations and support our recommendation for age-stratified and gender-sensitive management approaches. Our findings emphasize the clinical need for gastrointestinal risk assessment in elderly CVD patients. Targeted prevention and management strategies, especially in older males, may help reduce adverse outcomes and improve clinical outcomes in patients with coexisting cardiovascular and gastrointestinal conditions.

Nonetheless, this study has several limitations. First, it included participants aged 65–79, which may limit the applicability of the findings to older individuals with different risk profiles and health outcomes. Due to lower adherence to endoscopic screening in older age groups, the prevalence of PU in this population may be underestimated. Moreover, the diagnosis of PU was based on self-reported data without endoscopic confirmation, potentially resulting in misclassification. This may lead to under diagnosis or over-reporting, affecting prevalence estimates and outcome associations. In addition, the database lacked information on ulcer severity, such as Forrest classification, and lesion location, limiting risk stratification. Although acute PU complications such as bleeding and perforation are associated with high short-term mortality (33), detailed clinical data were unavailable. In a large study of 42,046 patients, 80.25% had ulcer-related complications, with bleeding and perforation mortality rates of 2.6 and 5.96% (34). It also did not contain data on concomitant medication use, such as NSAIDs or PPI therapy, which precluded assessment of drug-related ulcer risk and limited interpretation of relevant mechanisms. Furthermore, the cohort, while representative of older adults in China, may not be generalizable to other populations with differing ethnicities, healthcare access, or risk profiles. Finally, the conservative diagnostic criteria and handling of missing data enhanced internal validity but may have reduced sensitivity to detect modest associations. Future research should expand age coverage, incorporate more precise diagnostic tools, and further investigate sex- and age-specific mechanisms to improve risk prediction and guide individualized interventions in elderly PU patients.

## 5 Conclusion

This study among an elderly population demonstrates that PU does not significantly affect overall mortality or the prognosis of patients with CVD. While CVD was identified as a risk factor for PU, PU did not notably increase CVD risk. Nonetheless, older adults with PU remain at elevated risk of mortality, emphasizing the importance of early recognition and proactive management. These findings underscore the clinical need for integrated cardiovascular–gastrointestinal risk assessment, particularly in elderly and male patients. Future research should explore underlying mechanisms and long-term outcomes, and develop more refined, stratified intervention strategies to improve care for this vulnerable population.

## Data Availability

Publicly available datasets were analyzed in this study. This data can be found here: https://doi.org/10.18170/DVN/WBO7LK.

## References

[1] Forman DE Maurer MS Boyd C Brindis R Salive ME Horne FM. Multimorbidity in older adults with cardiovascular disease. J Am Coll Cardiol. (2018) 71:2149–61. doi: 10.1016/j.jacc.2018.03.022, PMID: 29747836 PMC6028235

[2] Voelker R. Peptic ulcer disease. JAMA. (2025) 333:917. doi: 10.1001/jama.2024.27195, PMID: 39946121

[3] Joshi DC Joshi N Kumar A Maheshwari S. Recent advances in molecular pathways and therapeutic implications for peptic ulcer management: a comprehensive review. Horm Metab Res. (2024) 56:615–24. doi: 10.1055/a-2256-6592 38467155

[4] Vakil N. Peptic ulcer disease: a review. JAMA. (2024) 332:1832–42. doi: 10.1001/jama.2024.19094, PMID: 39466269

[5] Zheng Y Xue M Cai Y Liao S Yang H Wang Z. Hospitalizations for peptic ulcer disease in China: current features and outcomes. J Gastroenterol Hepatol. (2020) 35:2122–30. doi: 10.1111/jgh.15119, PMID: 32452066

[6] Kang JY Tinto A Higham J Majeed A. Peptic ulceration in general practice in England and Wales 1994–98: period prevalence and drug management. Aliment Pharmacol Ther. (2002) 16:1067–74. doi: 10.1046/j.1365-2036.2002.01261.x 12030947

[7] Kavitt RT Lipowska AM Anyane-Yeboa A Gralnek IM. Diagnosis and treatment of peptic ulcer disease. Am J Med. (2019) 132:447–56. doi: 10.1016/j.amjmed.2018.12.009, PMID: 30611829

[8] Murray CJL Barber RM Foreman KJ Ozgoren AA Abd-Allah F Abera SF. Global, regional, and national disability-adjusted life years (DALYs) for 306 diseases and injuries and healthy life expectancy (HALE) for 188 countries, 1990–2013: quantifying the epidemiological transition. Lancet. (2015) 386:2145–91. doi: 10.1016/S0140-6736(15)61340-X 26321261 PMC4673910

[9] Jurgens CY Lee CS Aycock DM Masterson Creber R Denfeld QE DeVon HA. State of the science: the relevance of symptoms in cardiovascular disease and research: a scientific statement from the American Heart Association. Circulation. (2022) 146:e173–e184. Available online at: https://www.ahajournals.org/doi/10.1161/CIR.0000000000001089 35979825 10.1161/CIR.0000000000001089

[10] Liu M Meijer P Lam TM Timmermans EJ Grobbee DE Beulens JWJ. The built environment and cardiovascular disease: an umbrella review and meta-meta-analysis. Eur J Prev Cardiol. (2023) 30:1801–27. doi: 10.1093/eurjpc/zwad241, PMID: 37486178

[11] Roth GA Johnson C Abajobir A Abd-Allah F Abera SF Abyu G. Global, regional, and national burden of cardiovascular diseases for 10 causes, 1990 to 2015. J Am Coll Cardiol. (2017) 70:1–25. doi: 10.1016/j.jacc.2017.04.052, PMID: 28527533 PMC5491406

[12] Zhou M Wang H Zhu J Chen W Wang L Liu S. Cause-specific mortality for 240 causes in China during 1990–2013: a systematic subnational analysis for the global burden of disease study 2013. Lancet. (2016) 387:251–72. doi: 10.1016/S0140-6736(15)00551-6, PMID: 26510778

[13] Zhao D Liu J Wang M Zhang X Zhou M. Epidemiology of cardiovascular disease in China: current features and implications. Nat Rev Cardiol. (2019) 16:203–12. doi: 10.1038/s41569-018-0119-4 30467329

[14] Gosch M. Pharmakologische therapie kardiologischer erkrankungen im alter. Z Gerontol Geriat. (2022) 55:471–5. doi: 10.1007/s00391-022-02084-w, PMID: 35849160

[15] Ciumărnean L Milaciu MV Negrean V Orășan OH Vesa SC Sălăgean O. Cardiovascular risk factors and physical activity for the prevention of cardiovascular diseases in the elderly. Int J Environ Res Public Health. (2021) 19:207. doi: 10.3390/ijerph19010207 35010467 PMC8751147

[16] Wizenty J Koop PH Clusmann J Tacke F Trautwein C Schneider KM. Association of *Helicobacter pylori* positivity with risk of disease and mortality. Clin Transl Gastroenterol. (2023) 14:e00610. doi: 10.14309/ctg.0000000000000610, PMID: 37367296 PMC10522101

[17] Aggarwal K Singh S Singla A Kanagala SG Anamika F Singh B. Unveiling the silent intruder: *H. pylori’s* hidden link to ischemic heart disease. Cardiol Rev. (2024). doi: 10.1097/CRD.0000000000000686 38445894

[18] Ridker PM Luscher TF. Anti-inflammatory therapies for cardiovascular disease. Eur Heart J. (2014) 35:1782–91. doi: 10.1093/eurheartj/ehu203, PMID: 24864079 PMC4155455

[19] Chen J Sun Y Fu T Lu S Shi W Zhao J. Risk of incident cardiovascular disease among patients with gastrointestinal disorder: a prospective cohort study of 330 751 individuals. Eur Heart J Qual Care Clin Outcomes. (2024) 10:357–65. doi: 10.1093/ehjqcco/qcad059, PMID: 37777843

[20] Yi Z Poston Jr DL Vlosky DA Gu D. Introduction to the Chinese longitudinal Healthy Longevity Survey (CLHLS) In: Healthy longevity in China: demographic, socioeconomic, and psychological dimensions. Berlin, Germany: Springer (2008). 23–38.

[21] Li N Wang Z Bao Y Tang H Ma J Zheng Y. Analysis of disease burden and changing trend of peptic ulcer in China from 1990 to 2019 2023. Modern. Prev Med. (2023) 50:3090–53101. doi: 10.20043/j.cnki.MPM.202305375

[22] Safar ME Nilsson PM. Pulsatile hemodynamics and cardiovascular risk factors in very old patients: background, sex aspects and implications. J Hypertens. (2013) 31:848–57. doi: 10.1097/HJH.0b013e32835ed5b9, PMID: 23449020

[23] Ren J Jin X Li J Li R Gao Y Zhang J. The global burden of peptic ulcer disease in 204 countries and territories from 1990 to 2019: a systematic analysis for the global burden of disease study 2019. Int J Epidemiol. (2022) 51:1666–76. doi: 10.1093/ije/dyac033, PMID: 35234893

[24] Liuzzo G Patrono C. *Helicobacter pylori* eradication as a gastroprotective strategy in elderly aspirin-treated subjects: established facts and unanswered questions. Eur Heart J. (2023) 44:711–2. doi: 10.1093/eurheartj/ehac808, PMID: 36638779

[25] Smith A Contreras C Ko KH Chow J Dong X Tuo B. Gender-specific protection of estrogen against gastric acid-induced duodenal injury: stimulation of duodenal mucosal bicarbonate secretion. Endocrinology. (2008) 149:4554–66. doi: 10.1210/en.2007-1597 18499763 PMC2553385

[26] Duarte GJ Lopez J Sosa F Molina G Shaban M Mark J. Proton pump inhibitors and cardiovascular risk: a critical review. Futur Cardiol. (2024) 20:779–94. doi: 10.1080/14796678.2024.2412910 PMC11622795 39466134

[27] Sun L Zheng H Qiu M Hao S Liu X Zhu X. *Helicobacter pylori* infection and risk of cardiovascular disease. Helicobacter. (2023) 28:e12967. doi: 10.1111/hel.12967, PMID: 36974892

[28] Wu H Wei M Li N Lu Q Shrestha SM Tan J. Clopidogrel-induced gastric injury in rats is attenuated by stable gastric pentadecapeptide BPC 157. Drug Des Devel Ther. (2020) 14:5599–610. doi: 10.2147/DDDT.S284163, PMID: 33376304 PMC7763470

[29] Chen J. GPD2 inhibition impairs coagulation function via ROS/NF-κB/P2Y12 pathway. Cell Mol Biol Lett. (2025) 30:84. doi: 10.1186/s11658-025-00759-x 40682019 PMC12273321

[30] Huang J Liao F Tang J Shu X. Risk factors for gastrointestinal bleeding in patients with cerebral infarction after dual antiplatelet therapy. Clin Neurol Neurosurg. (2023) 231:107802. doi: 10.1016/j.clineuro.2023.107802, PMID: 37295199

[31] Niccoli G Franceschi F Cosentino N Giupponi B De Marco G Merra G. Coronary atherosclerotic burden in patients with infection by CagA-positive strains of *Helicobacter pylori*. Coron Artery Dis. (2010) 21:217–21. doi: 10.1097/MCA.0b013e3283399f36, PMID: 20389238

[32] Dahal K Sharma SP Kaur J Anderson BJ Singh G. Efficacy and safety of proton pump inhibitors in the long-term aspirin users: a meta-analysis of randomized controlled trials. Am J Ther. (2017) 24:e559–69. doi: 10.1097/MJT.0000000000000637, PMID: 28763306

[33] Cazacu SM Surlin VM Rogoveanu I Goganau A Iovanescu VF Ghinea AL. Trends for admission and mortality in peptic ulcers at a tertiary referral hospital during the 2017–2021 period. Chirurgia (Bucur). (2024) 119:404–16. doi: 10.21614/chirurgia.2923, PMID: 39250610

[34] Havens JM Castillo-Angeles M Nitzschke SL Salim A. Disparities in peptic ulcer disease: a nationwide study. Am J Surg. (2018) 216:1127–8. doi: 10.1016/j.amjsurg.2018.08.025, PMID: 30224069

